# Post-Secondary Education Outcomes for Individuals with Intellectual and Developmental Disabilities: Self-Determination, Independent Living, Employment, and the Impact of COVID-19

**DOI:** 10.3390/bs13100832

**Published:** 2023-10-11

**Authors:** Dalun Zhang, Eric Roberts, Robert Maddalozzo, Yi-Fan Li, Meagan Orsag, Tracy Glass

**Affiliations:** 1 Department of Educational Psychology, Center on Disability and Development, Texas A&M University, College Station, TX 77843, USA; dalun@tamu.edu (D.Z.); robe1330@msu.edu (E.R.); robert.maddalozzo@dodea.edu (R.M.); meaganorsag@tamu.edu (M.O.); tglass@tamu.edu (T.G.); 2 Department of Interdisciplinary Learning and Teaching, University of Texas-San Antonio, San Antonio, TX 78249, USA

**Keywords:** post-secondary education, intellectual and developmental disabilities, employment, self-determination, COVID-19

## Abstract

Post-secondary education (PSE) plays an important role in preparing individuals with an intellectual and developmental disability (IDD) to gain employment and independent living. Despite the recent increase in PSE programs, however, there is a lack of research examining outcomes of individuals with IDD who have attended a PSE program. This study examined three years of data from students who participated in one PSE program that focuses on the acquisition of skills regarding self-determination, employment, and independent living. We analyzed the outcome data of program graduates regarding their acquisition of skills in employment and self-determination, as well as follow-up data on employment. It was found that participation in the program increased self-determination and post-secondary readiness. Our findings also indicated that the COVID-19 pandemic impacted the self-determination scores of participants and their employment outcomes.

## 1. Introduction

### 1.1. Post-Secondary Education Programs

Post-secondary education (PSE) is an important option for individuals with an intellectual and developmental disability (IDD) to continue their learning. PSE provides a variety of experiences for these individuals such as coursework, independent living, social interactions, and many work-related activities. As a result of high demand, there has been a steady increase in the number of PSE programs for students with IDD. Think College [1] reported 323 PSE programs in 2023 for students with IDD, and more programs may be established and expanded in the future. PSE serves as a bridge to connect education to a paid job so that individuals with IDD can receive training for essential job skills [2]. Thoma et al. [3] described that the primary reasons for students with IDD to attend a PSE program include receiving instructions in a more inclusive setting and improving employment outcomes. In a survey study of 149 PSE programs for students with intellectual disability (ID) across 39 states, Grigal et al. [4] found that employment was one of the main focuses of the programs. The types of employment services included job shadowing and internships, with integrated competitive employment as one of the expected outcomes. Families also considered employment-focused PSE programs to be beneficial for young adults with disabilities. The findings of a study by Griffin et al. [5] on family attitudes towards PSE programs suggested that families believe PSE programs put more emphasis on employment preparation, leading to improved employment opportunities for their children. A variety of PSE programs thrive to prepare individuals with disabilities for employment. As the required skills for employment continue to evolve in the 21st century, there is a need to provide up-to-date career training for individuals with disabilities.

### 1.2. Existing Post-Secondary Education Programs Models

Different PSE program models may have unique missions and training focuses for students with disabilities. Stodden and Whelley [6] identified three types of PSE program models: the substantially separate model, the mixed program model, and the individualized support model. The substantially separate model means students in a PSE program are separate from other college students. Its curriculum is specifically designed for the program students. The mixed program model incorporates not only a specially designed curriculum but also regular courses meant for matriculated students. This model gives students the chance to interact and study with matriculated peers. The individualized support model ensures that students in a PSE program receive student-centered and individualized services, which include accommodations. These tailored services assist students in participating in a range of college courses within an inclusive environment. Historically, post-secondary opportunities at the college level have transitioned from segregation to inclusion in recent years. As a result, students can now experience diverse training that fosters connections to community-based settings [7].

Another way to identify the models of PSE programs for students with IDD is through a taxonomy provided by McEathron et al. [8]. The taxonomy outlines four domains and 16 components to categorize PSE programs. The four domains include: the Organizational Domain (program and institutional characteristics), Admissions Domain (program criteria for student recruitment), Support Domain (support provided for students), and Pedagogical Domain (course or credit elements). Among these domains, the Pedagogical Domain specifically outlines the different components of courses, such as academic, vocational, independent living, and social components. Although the taxonomy identified a variety of PSE programs that prepare students for their career development, McEathron and their colleagues stressed that the vocational component in PSE programs may need to undergo a change in the future because industries and the skills needed for jobs are more different nowadays compared to how they were a few years ago. Additionally, more programs are pursuing a process of providing certain employment-related certificates, such as Direct Support Professional and Child Development Associate, for students with disabilities.

### 1.3. Post-Secondary Education Outcomes

#### 1.3.1. Employment

Recent studies have shown that post-secondary educational experiences and achievements correlate with better employment outcomes. This includes working longer hours, earning a higher hourly wage, and securing positions in a variety of professions. For example, by analyzing data from state-federal vocational rehabilitation services, Cimera et al. [9] found that individuals with some post-secondary education experience were more likely to be in employment, work more hours, earn a better hourly wage, and have jobs in a wider variety of vocations compared to their counterparts (those without any post-secondary education experience). Similar results can also be found in Miller et al. [10] and Whittenburg et al. [11]. Both of these research studies also indicated that developing employment training opportunities in PSE for young adults with disabilities can help such individuals to explore specific work skills in a wider range of fields. On the contrary, individuals with IDD who did not have some post-secondary education experiences had less earnings [10]. Such individuals with less educational experience were also less likely to be employed and more likely to work fewer weekly hours [11]. Grigal et al. [12] stated that only 19% of adults with IDD had a paid job in the community in the period 2018–2019. These facts highlight an imperative need for individuals with IDD to attend PSE programs and receive employment-focused training.

Focusing on PSE programs designed for increasing the employability of individuals with ID, Moore and Schelling [13] demonstrated another notable finding related to employment outcomes. They surveyed 26 graduates of two PSE programs and interviewed program administrators. One of the programs was considered to have used the individualized support model; the other one was considered to have adopted the substantially separate model. Graduates of both programs showed encouraging employment outcomes, and those who received inclusive training experiences were more likely to work in competitive employment environments. The study results also revealed positive employment outcomes for students with significant disabilities who attended a PSE program.

#### 1.3.2. Independent Living

In addition to employment outcomes, research has shown positive outcomes in other areas for those who have attended PSE programs for students with disabilities. Ross et al. [14] followed up 125 individuals with IDD for ten years from the time of graduation from a PSE program. The program provided on-campus housing for students, typical collegiate classes, and specialized classes that focused on academic skill development, independent living skills, career preparation, and transition planning. Using a survey to evaluate students’ employability, independent living skills, and other areas of personal growth, the findings revealed that those who graduated from PSE programs were more likely to b employed and that they earned more compared to their peers with IDD who had not participated in any PSE programs. The study also indicated enhanced outcomes regarding independent living, such as achieving financial independence and using public transportation. The majority of the participants lived in an apartment either alone or with a spouse or roommate. Prohn et al. [15] examined the independent living outcomes of a PSE program for six students with ID. The participants in the program received full inclusive experiences with the support from individual tutoring on specific skills. Utilizing the Scale of Independent Behaviors-Revised [16] and the Support Intensity Scale [17] as evaluation tools, the results indicated that the program enhanced independence and reduced support needs. This suggests that participation in the program bolstered students’ ability to function independently. While the study’s sample size was limited to six participants, the initial evidence showed that the PSE program provided a conducive environment for students to learn independence.

#### 1.3.3. Self-Determination

Self-determination is one of the most important skills for students with IDD to learn during their transition after high schools. Wehmeyer [18] defined self-determination as “acting as the primary causal agent in one’s life and making choices and decisions regarding one’s quality of life free from undue external influence or interference” (p. 632). Individuals with self-determination can take responsibility and steer their lives towards a personally satisfying direction. Self-determination has been identified as a predictor that influences students’ enrollment in and their completion of post-secondary education programs [19,20]. However, PSE outcomes on self-determination for students with IDD have not been extensively covered in the existing literature. Getzel [21] stated that PSE environments can support students with IDD in cultivating self-determination. Getzel [21] also underscored that more research studies are needed to show evidence in the PSE environments that support self-determination. This highlights that research studies should investigate whether a post-secondary education program can enhance the self-determination of students with IDD.

### 1.4. The Impact of the COVID-19 Pandemic on Students’ Learning Experiences

The COVID-19 pandemic suddenly changed both learning and teaching in an unexpected way. Although remote or distance learning is not a new learning and teaching method, the abrupt shift to remote/online learning in spring 2020 still affected students’ learning experiences [22]. The abrupt shift to remote/online learning constituted a crisis for instruction and learning. A few research studies investigated the learning experiences among college students during this time (e.g., [23,24,25]). These studies revealed challenges related to the abrupt shift to remote/online learning, such as inadequate internet service and inadequate electronic devices to facilitate online learning. For students with IDD in a PSE program, Spencer et al. [26] explored how students with IDD navigated the COVID-19 pandemic-induced disruption and the challenges they faced. The study revealed that students in the PSE program demonstrated more resilience to navigate the disruption. However, the study also demonstrated that students faced greater challenges in looking for a job during this time because of business closures and social distancing guidelines. Given the limited research on how the COVID-19 pandemic affected students with IDD, more research should explore its impact on these students within PSE programs.

### 1.5. Current Study

The purpose of this study was to examine the effectiveness of the Post-secondary Access and Training in Human Services (PATHS), an inclusive and career-focused PSE program. We identified that the PATHS program adopts the substantially separate model. Nonetheless, the students enrolled in the program had opportunities to engage in inclusive on-campus activities. Although previous studies have shown some evidence of improved employment outcomes through PSE, the current literature is sparse regarding PSE programs specifically designed for students with IDD. There is a need for more research studies to understand how a specially designed PSE program impacts learning outcomes for people with IDD [4,13].

The focus of this study was on evaluating the effectiveness of PATHS in preparing individuals with IDD for careers that promote self-determination and employment skills, as well as on post-graduation outcomes. The student cohorts targeted for this study graduated from the program in 2018, 2019, or 2020. Our hypothesis is that different cohorts consisted of students from diverse disabilities and cultural backgrounds. Thus, these distinct cohorts may yield differing outcomes.

In 2020, the COVID-19 pandemic swept across the globe, changing the way people lived and worked. As previously mentioned, the sudden shift to online learning influenced the learning experiences of students. Some studies, such as the one conducted by Sinclair et al. [27], have indicated that the pandemic significantly changed people’s work lives, as many experienced reduced working hours and job losses. Therefore, this study also examined the impact of the COVID-19 pandemic on program offerings, student learning, and post-graduation outcomes. The research questions included: (1) What effect does participation in the PATHS program have on students’ self-determination skills? (2) What effect does participation in the PATHS program have on students’ post-secondary readiness skills? (3) Are there any differences among disability type, gender, and ethnicity in terms of their skills acquisition and post-graduation outcomes? (4) What effect does participation in the PATHS program have on students’ post-graduation employment outcomes? The impact of the COVID-19 pandemic on students’ learning and post-graduation outcomes were included in Questions 1, 2, and 4.

## 2. Methods

The researchers of this study formed a team (hereafter, we) to analyze the data. We employed a quantitative correlational research design, focusing on the effectiveness of the PATHS program by examining its impact on self-determination and post-secondary readiness skills. The correlational research design helps researchers to understand an association between variables. This study was approved by the university’s institutional review board.

### 2.1. The PATHS Program

The PATHS program is a post-secondary education program offered at a research-intensive university in the Southern United States. Each year, the PATHS program accepts 15–20 students for the program. Students in the program can choose one of two career tracks: The Direct Support Professional (DSP) or the Child Care Professional (CCP) certification programs. Both these professional roles have recruitment and retention issues. One of the retention challenges is training. The two professionals should be well trained to provide high-quality services for individuals with disabilities and young children. However, many DSPs and CCPs choose to leave their post due to inadequate training leaving them unsure of how to provide appropriate support for individuals [28,29]. In this context, the mission of the PATHS program is to train individuals with IDD to become service providers who offer high-quality services to individuals with disabilities and young children. The following paragraphs contain additional information about the program:

(1) Admission Criteria:

Applicants must be 18 years or older when the program begins (there is no age limit). Applicants must be their own guardian. Applicants must have graduated from high school or have completed their General Educational Development tests. Applicants must be able to pass a criminal background check.

(2) Admission Process:

The submission window for applications usually opens in November and closes in February. Online applications and supplemental documentation must be completed and submitted by the deadline. Applications are scored according to a rubric to determine who will move forward to the interview stage. Interviews are scored according to a rubric to determine who will be accepted on to the program. Applicants who demonstrate a passion for human services and have experience working with individuals with disabilities and young children are more likely to move forward to the interview stage and receive acceptance from the program administrators.

(3) Course of Study:

The PATHS program adopts a cohort model. The PATHS program is designed to span one year with three semesters (summer semester, fall semester, and spring semester). The program provides students with multiple opportunities for both direct and indirect training. Students receive instructions in a variety of areas, including independent living/life skills, employment, and self-determination. The self-determination and employment skills training curriculum are presented as direct instruction activities/classes that are embedded across all other courses and activities. Students also complete over 160 work-based learning hours and receive direct and indirect support while completing these experiences. During their time in the PATHS program, students live off campus and receive direct and indirect independent living support. In the off-campus living environment, they are housed with other students (not necessarily with PATHS students). The PATHS program also offers students opportunities to be active on campus and engage in curricular and extracurricular activities. The curricular/extracurricular activities could be activities emanating from on-campus organizations and community events. Students are also encouraged to attend specially designed classes and practice independent living skills in a dorm-based environment while they simultaneously receive focused instructions on career skills and self-determination.

(4) Instructor Information:

The program’s instructors have experience working with individuals with disabilities and the ability to specially design course materials to teach students. Each course has one instructor and one to two instructional aides. The instructional aides are present in all classes to ensure students receive adequate support. In addition, students are assigned advisors for peer mentoring and support. Students are required to meet with them at least twice weekly. Support is phased out as the students become more independent.

### 2.2. Sample

The student cohorts targeted for this study graduated from the program in 2018, 2019, or 2020 (See Table 1). The graduation rates for the three cohorts are 95%, 96%, and 100%, respectively. The 2017–2018 cohort included nine females and seven males (seven White, three Hispanic, three African American, one Asian, and two more than one race). The distribution of their primary disabilities was as follows: four with ID, four with other health impairment (OHI), two students with visual impairment (VI), two students with traumatic brain injury (TBI), two with specific learning disability (SLD), and two with emotional disturbance (ED).

The 2018–2019 cohort included 14 female and three male students. Of the 17 students, 11 were White, 3 were Hispanic, 1 was Asian, 1 student belonged to more than one race, and 1 student’s race was listed as “other.” The distribution of their primary disabilities was as follows: five students with ID, four students with OHI, two students with VI, two students with hearing impairment (HI), two students with autism spectrum disorder (ASD), and one student with SLD. One student had a disability that was not documented in the IEP from the school.

The 2019–2020 cohort included 10 females and 5 males. Ten students were White, three Hispanic, one Asian, and one student more than one race. The distribution of their primary disabilities was as follows: eight students with ID, two students with OHI, two students with SLD, one student with VI, one student with HI, and one student with ASD.

### 2.3. Assessment

This study used two instruments, the AIR Self-Determination Scale and Casey Post-secondary or Training Assessment, to assess students’ level of self-determination and independent living skills. The aim of using the AIR Self-Determination Scale was to measure an individual’s capacity and opportunities across four domains: autonomy, self-regulation, psychological empowerment, and self-realization. This instrument also provides an overall self-determination score. It has 30 items, 18 are related to capacity; 12 are related to opportunity. Capacity was defined as the perceptions of ability to cause change, and Opportunity was defined as perceptions of chances to apply knowledge and abilities. Higher scores correspond to increased capacity or opportunity.

Reliability information has been identified as correlations above 0.91 in alternative-item analysis, 0.95 in split test analysis, and 0.74 in test–retest analysis [30]. Validity measures accounted for 74% of variance in the assessment of the measures [30]. The assessment included student, parent, and educator forms (offered in both English and Spanish). This study used the student form written in English to collect data. The assessment included four areas of questions: “Things I do”, “How I feel”, “What happens at school”, and “What happens at home.” The AIR has been used in many other studies regarding self-determination.

The Casey Post-secondary or Training Assessment is a toolkit-based measure that is meant to monitor preparedness and does not report its reliability or validity factors. As this was only used to show growth in preparedness in relation to specific items and the changes were consistent in administration, the validity of the measure on the identified areas or the reliability of the scores should not be impacted. The assessment focuses on the areas of daily living, relationships, work/study, community resources, money management, computer literacy, civic engagement, and navigating the welfare system. As the PATHS program under study had specific areas of instruction and support, the instruction team of the PATHS programs found that it was challenging for students to complete all areas of the assignment; thus, the team decided to eliminate areas that were not applicable to the students (e.g., navigating the welfare system). Students only completed the following areas of the assessment: (1) career and education planning, (2) study and technology, (3) motivation and participation, (4) school or program, (5) supports, (6) health, (7) foster care issues, and (8) financial aid and budgeting. Higher scores correspond to increased skills.

Both the AIR and Casey assessments were put into Qualtrics (Qualtrics, Provo, UT, USA) for scoring, a system that is familiar to all PATHS Program students. Assessments were administered in an assessment course. In the course, students focused on completing the assessments. The instruction team in the program was available to provide additional support for students who may need someone to read the assessment questions to them. A total of four assessments were administered throughout the program, each different time points: (1) a pre-assessment at the start of the fall term, (2) a post-assessment at the end of the fall term, (3) a pre-assessment at the start of the spring term, and (4) a post-assessment at the end of the spring term (when students were expected to graduate).

A short follow-up survey was created to collect data on post-graduation employment and the possible impact of the COVID-19 pandemic. The survey was conducted in a one-time point of collection manner in 2020 during the pandemic to follow up the three cohorts who graduated from the program in 2018, 2019, or 2020. Graduates who completed the survey must have included their name and other identifiable information in order to be considered in the study sample. We created the survey questions based on regular team meetings and consulted relevant studies (e.g., Moore and Schelling, 2015). Following multiple team discussions, we refined the questions to enhance their clarity and frame them in a quantitative nature. The focus of the questions were as follows: (a) whether the graduate was employed immediately after graduating from the PATHS program (e.g., they started their job within a month of graduating from the PATHS program), (b) whether they were still employed at the time of data collection, (c) whether they were employed in the area of their certification, (d) how much they were paid, and (e) how long/often they were employed and worked. Participants could also opt to provide more robust explanations of their answers. The pandemic-related questions were qualitative in nature, focusing on how they perceived the impact of the pandemic on their employability and ability to find a job. We invited graduates to complete the follow-up survey by sending out emails. Additionally, because some graduates were followers of the PATHS social media page, we posted the survey on the social media page as well. Graduates could view a flyer on the social media page and click on a link that would direct them to the follow-up survey.

### 2.4. Data Analysis

Before we conducted data analysis, we transferred the data from Qualtrics to SPSS. The independent variables were the testing periods, disability type, gender, ethnicity, and cohort. The dependent variables that were measured by the surveys and interviews were overall self-determination scores, capacity scores, opportunity scores, Casey Post-secondary or Training Assessment Scores, employment rates, and level of employment. In order to do this, three cohorts were evaluated via pre-assessments, continual progress monitoring, and post-assessments. An additional follow-up survey was conducted with graduates to assess post-graduation employment, as well as the impact of COVID-19 had on their employment opportunities.

Data were analyzed through repeated-measure regression analyses. Repeated-measure regression analyses were employed because the dependent variables were on a continuous scale; there were more than two independent variables, there was a linear relationship between variables, and there were no significant outliers. This approach allowed for an evaluation of statistical significance regarding the relationships between the independent and dependent variables over the course of the PATHS program, as well as an analysis between each iteration of the assessment phase.

Our analysis of post-program employment was limited by the number of participants (*N* = 12). Due to the limited number of participants, we descriptively summarized the survey findings. The descriptive summary provided an understanding of how graduates perceived the impact of the PATHS Program on their employment opportunities and how the training prepared them for future employment opportunities.

## 3. Results

### 3.1. Research Question 1: What Effect Does Participation in the PATHS Program Have on Students’ Self-Determination Skills?

#### 3.1.1. The AIR Self-Determination Assessment: Overall Scores

**Figure 1:**
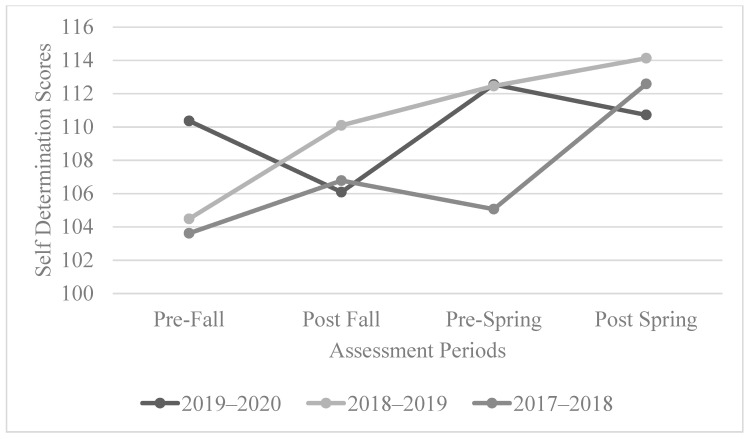
The overall trend of self-determination scores.

The overall self-determination scores across the 2017–2018 and 2018–2019 cohorts showed a positive trend from the start of the fall term to the end of the spring term. The positive trend demonstrated that the overall level of self-determination had grown during the year across participants. However, for the 2019–2020 cohort, there was a dramatic decrease between the start of the fall term and end of the fall term and then an increase from the end of the fall term into the early spring term. However, the scores again dropped as schools were forced to quickly adopt remote learning environments due to the worldwide pandemic. See Figure 1 for the overall trend of the self-determination scores.

We also ran statistical analyses by using repeated measures to examine the level changes for each cohort. The overall self-determination scores over the course of the program for the 2017–2018 cohort were found to be statistically significant (*p* = 0.001 < 0.05), especially when comparing between those for the pre-fall test and pre-spring test (*p* = 0.044 < 0.05) and the pre-spring and post-spring test (*p* = 0.001 < 0.05). However, the overall self-determination scores for the 2018–2019 cohort were not statistically significant over the four time periods. The results between the pre-fall and post-spring tests were not statistically significant, although there was a constant positive trend in self-determination scores for this cohort (Figure 1).

For the 2019–2020 cohort, the scores were not statistically significant. This lack of significance is likely associated with the significant drop between the pre-spring and post-spring scores (associated with the start of the pandemic changes). However, the overall trend was still positive, but additional data points are needed to better understand the impact of the changes that took place in the spring of 2020.

The overall self-determination scores show a positive trend, but they do not identify the specific areas of self-determination. To do this, an analysis of the capacity and opportunity scores is necessary. This analysis allows for a better understanding of the participants’ experiences and the areas of self-determination impacted by participation in the PATHS program.

#### 3.1.2. The AIR Self-Determination Assessment: Capacity

**Figure 2:**
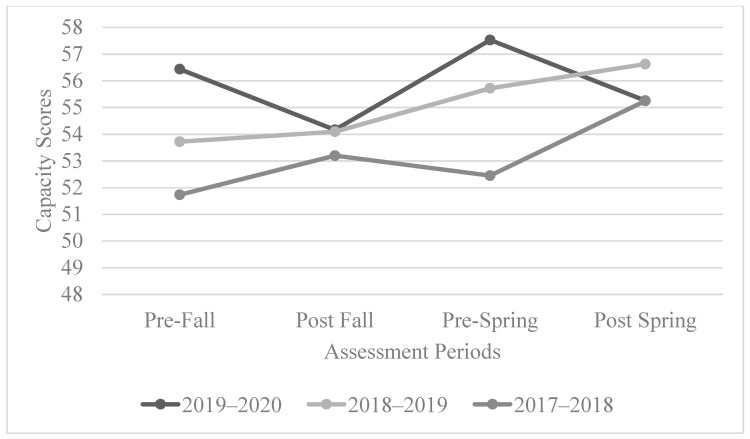
The overall trend of capacity scores.

The capacity scores for the 2017–2018 cohorts showed positive growth from the start of the fall term to the end of the spring term. For the 2018–2019 cohorts, there was a positive growth trend from the start of the fall term to the end of the fall term. After the end of the fall term, the scores were not only maintained but also increased towards the end of the spring term. For the 2019–2020 cohorts, there was a decrease between the start of the fall term and end of the fall term and then an increase from the end of the fall term into the early spring term. The scores again decreased from the early spring term to the end of spring term. See Figure 2 for the overall trend of the capacity scores.

The results of the statistical analysis showed that for the 2017–2018 cohort, capacity scores over the course of the program were statistically significant (*p =* 0.006 < 0.05). For the 2018–2019 cohort, there were no statistically significant results, although, as previously mentioned, there was a positive trend. Regarding the 2019–2020 cohort, their scores were not found to be statistically significant, though the decrease did align with the beginning of the COVID-19 pandemic. While not considered statistically significant for the 2018–2019 and 2019–2020 cohorts, across all three cohorts, there was a positive trend in capacity scores.

#### 3.1.3. The AIR Self-Determination Assessment: Opportunity

**Figure 3:**
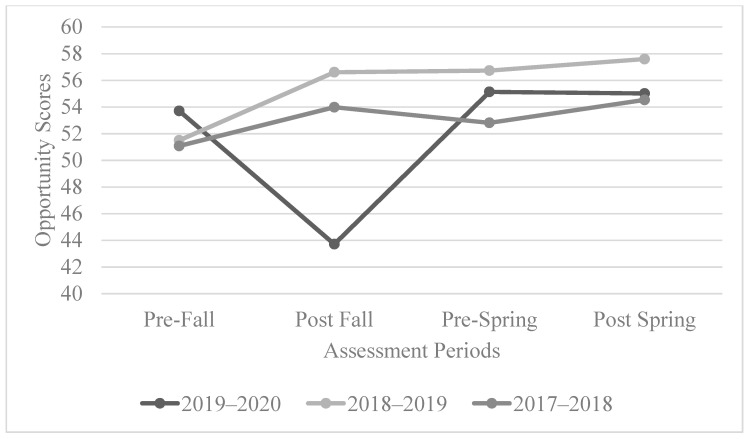
The overall trend of opportunity scores.

The opportunity scores for the 2017–2018 cohort had a positive trend. Similarly, the 2018–2019 cohorts had an overall positive trend, but the trend was much less pronounced between the fall and spring. For the 2019–2020 cohort, a decrease was found from pre-fall to post-fall, and the scores increased sharply from post-fall to pre-spring. See Figure 3 for the overall trend of the opportunity scores.

The results of the statistical analysis of the opportunity scores for the 2017–2018 cohort showed that they were statistically significant (*p* = 0.001 < 0.05). These score differences were also statistically significant between the pre-fall and post-spring assessments (*p* = 0.04 < 0.05). However, there were no statistically significant results regarding the opportunity scores for the 2018–2019 cohorts, though there was a positive trend overall. For the 2019–2020 cohort, these scores were not found to be statistically significant.

### 3.2. Research Question 2: What Effect Does Participation in the PATHS Program Have on Students’ Post-secondary Readiness Skills?

#### Casey Post-Secondary or Training Scale

**Figure 4:**
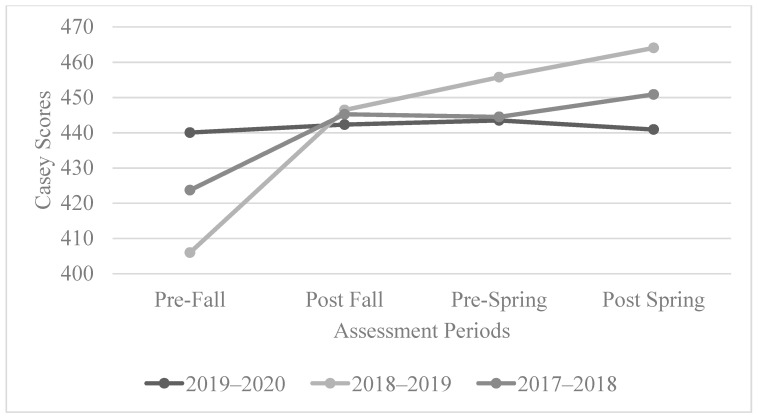
The overall trend of the Casey Post-secondary or Training Scale scores.

The participant scores on the Casey Post-secondary or Training Scale for the 2017–2018 cohort had a positive trend, with the greatest points of growth being between the pre-fall and post-fall assessments and the pre-spring and post-spring scores. Similar to the 2017–2018 cohort, there was a positive trend for the 2018–2019 cohort. While the growth between the pre-fall and post-fall scores was more substantial than through the rest of the year, the growth between the post-fall and post-spring scores was consistent and positive. Similar to the 2017–2018 and 2018–2019 cohorts, there was an initial positive trend for the 2019–2020 cohort’s Casey scores; however, these scores dropped by the post-spring assessment, and this is consistent with the results found on the AIR assessments. See Figure 4 for the overall trend of the Casey Post-secondary or Training Scale scores.

Over time, the participant scores for the 2017–2018 cohort were statistically significant (*p* = 0.033 < 0.05). The participant scores for the 2018–2019 cohort were also found to be statistically significant (*p* = 0.008 < 0.05); specifically, the scores between the pre-fall and post-spring period were found to be statistically significant (*p* = 0.04 < 0.05). However, the scores for the 2019–2020 cohorts were not found to be statistically significant.

### 3.3. Research Question 3: Are There any Differences among Disability Type, Gender, and Ethnicity in Terms of Their Skills Acquisition and Post-Graduation Outcomes?

#### 3.3.1. Self-Determination scores: AIR and Disability

The self-determination scores based on participant disabilities for the 2017–2018 cohort were found to be statistically significant (*p* = 0.001 < 0.05). Participants with ED scored higher than participants with ID, OHI, SLD, TBI, or VI, and participants with SLD scored higher than participants with ID, TBI, or VI. The self-determination scores based on participant disabilities for the 2018–2019 cohort were not found to be statistically significant. However, according to the single comparisons, participants with ID were found to score higher than participants with ASD, and participants with SLD were found to score higher than participants with ASD, ID, OHI, and VI. The self-determination scores based on participant disabilities for the 2019–2020 cohort were not found to be statistically significant in their relation to growth over the program or between groups. However, participants with ASD did score higher than participants with OHI, ID, or VI. Participants with SLD had higher self-determination scores than participants with ASD, ID, OHI, or VI.

#### 3.3.2. The Scores of the Casey Post-Secondary or Training Scale and Disability

For the 2017–2018 cohort, the life skill scores were not statistically significant indicators of participant scores, though participants with ED scored higher than students with ID, TBI, or VI as their identified primary disabilities. Participants with SLD were found to score higher than students with ID or TBI. The 2018–2019 cohort scores, when considering disability as a variable, were found to be statistically significant (*p* = 0.029 < 0.05). Participants with VI were found to have higher scores than participants with ASD, ID, or OHI. Participants with SLD scored higher than participants with ASD, ID, OHI, or VI. The scores based on disability for the 2019–2020 cohort were found to be statistically significant (*p* = 0.041 < 0.05); individuals with SLD were found to score higher than participants with ASD, OHI, ID, or VI.

#### 3.3.3. Self-Determination Scores: AIR and Gender

For the 2017–2018 cohort, the self-determination scores based on gender were not found to be statistically significant. However, for the single comparison, males had statistically significant growth in their self-determination scores compared to females over the course of the program (*p* = 0.015 < 0.05). The participant scores based on gender for the 2018–2019 cohort were not found to be statistically significant, though females did score higher than males over the course of the program. For the 2019–2020 cohort, the self-determination scores based on gender were not found to be statistically significant, although the self-determination scores for females were found to be higher than that of males.

#### 3.3.4. The Scores of Casey Post-Secondary or Training Scale and Gender

For the 2017–2018 cohort, their scores based on gender were not statistically significant. However, while not statistically significant, males were found to score higher than females. The 2018–2019 cohort scores were not statistically significant, but females scored higher than males. The 2019–2020 cohort scores were not found to be statistically significant, but females scored higher than males.

Gender was not found to significantly impact self-determination or life skill scores over the course of the PATHS program. However, there were substantial differences in self-determination scores based on gender, with participants who identified as female scoring higher than those who identified as male in the 2018–2019 and 2019–2020 cohorts.

#### 3.3.5. Self-Determination Scores: AIR and Ethnicity

For the 2017–2018 cohort, their self-determination scores based on ethnicity were found to be statistically significant (*p* = 0.001 < 0.05). Participants who identified as Asian statistically scored higher than participants who identified as more than one race (*p* = 0.006 < 0.05). Participants who identified as Black also scored higher than participants who identified as Asian, more than one race, or White. The relationship between participants who identified as Black and those who identified as more than one race was statistically significant (*p* = 0.006 < 0.05). Participants who identified as White scored higher than participants who identified as Asian and those who identified as more than one race (*p* = 0.006 < 0.05). Ethnicity was not found to be a statistically significant indicator of growth for the 2018–2019 cohort. However, participants who identified as more than one race scored higher than participants who identified as Asian, and participants who identified as White scored higher than participants who identified as Asian. For the 2019–2020 cohort, self-determination scores based on ethnicity were not found to be statistically significant indicators of growth in the program. However, participants who identified as Black scored higher than any other group (White, American Indian, or Alaskan Native, or more than one race) and participants who identified as White had higher scores than participants who identified as American Indian, Alaskan Native, or as more than one race.

#### 3.3.6. The Scores of Casey Post-Secondary or Training Scale and Ethnicity

For the 2017–2018 cohort, their life skill scores based on race were not found to be statistically significant. However, participants who identified as Asian scored higher than any other population, participants who identified as Black scored higher than participants who identified as more than one race or did not identify, and participants who identified as White scored higher than participants who identified as Black, did not identify, or identified as more than one race. While the scores for the 2018–2019 cohort were not statistically significant, participants who identified as more than one race scored higher than any other participants. Participants who identified as White also scored higher than participants who identified as Asian. The scores based on ethnicity, for the 2019–2020 cohort, were not found to be statistically significant. Participants who identified as Black scored higher than any other participant group, and participants who identified as White scored higher than participants who identified as American Indian or Alaskan Native and participants who identified as more than one race.

Ethnicity was not a statistically significant indicator of growth over the course of the program, although self-determination scores based on ethnicity were found to be statistically significant for the 2017–2018 cohort. However, it is clear that there was a relationship between ethnicity and self-determination scores or life skill scores. Participants who identified as Black or White had the highest self-determination or life skills scores.

### 3.4. Research Question 4: What Effect Does Participation in the PATHS Program Have on Students’ Post-Graduation Employment Outcomes?

#### 3.4.1. Post-Graduation Employment

Of the 12 graduates who responded to the PATHS Research Team’s outcomes interview, 8 (66.7%) had been employed after graduating from the PATHS program. Of these eight (66.7%) graduates who were employed, five graduates were employed in areas related to their PATHS certification (41.6%). Five of the graduates were employed within three months of graduation, one was employed within 5 months, and the other two opted not to answer this question. Of the employed graduates who responded about hours worked, three (25%) worked more than 30 h a week and one (8.3%) worked between 10 and 30 h a week. Of the respondents who answered questions about their wages, one (8.3%) graduate earned $7.25 an hour, four (33.3%) graduates earned between $9 and $10 an hour, and one (8.3%) graduate earned 12 dollars an hour; the minimum wage in the area was $7.25.

#### 3.4.2. The Impact of the COVID-19 Pandemic on Graduate Employment

Of the 12 respondents, 6 (50%) graduates identified that the pandemic impacted their employment opportunities. One graduate noted that they got their job while still in the PATHS Program, but it was not in their preferred job area, education, due to the pandemic inducing school closures. This graduate also noted that their hours were adversely impacted by the pandemic. The impact of the pandemic on hours worked was noted by another employed graduate, but they were able to gain employment in their job field (education). This was similar to another graduate employed in education, who noted that the pandemic impacted their role in the school due to the fact that they were not on campus. The other three graduates who noted that the pandemic was impacting their employment were not currently/had not been employed. These graduates noted that the pandemic was making it difficult to find employment opportunities in their area, and two were in education programs. One of these graduates noted that the pandemic was also making it difficult to even continue to do volunteer work in their community.

## 4. Discussion

The purpose of this study was to explore the effectiveness of the PATHS Program. In order to do this, student scores in self-determination and post-secondary readiness were evaluated and analyzed in relation to the study research questions. The results showed that the self-determination and post-secondary readiness scores for PATHS students had a positive trend across all three cohorts. These findings align with previous studies on similar topics. For example, Ross et al. [14] found that individuals with IDD who graduated from PSE programs were more likely to live and function independently. Prohn et al. [15] also demonstrated that individuals with IDD who participated in the PSE program had greater independent living skills. In the present study, participants in the PATHS program had to live in an off-campus dormitory with program peers. They learned to use the bus system for transportation to commute to campus and carry out grocery shopping.

For self-determination, as Getzel [21] stated, PSE environments are conducive to the cultivation of students’ self-determination. In PSE, great self-discipline is required. While students have more freedom, they also face more distractions and temptations. Therefore, students in PSE must learn self-management to balance their coursework and personal life [31,32]. For individuals with IDD, learning self-determination is crucial. Many individuals with IDD face challenges in monitoring and regulating their learning [33]. They may also experience a hard time in organizing what they have learned in class. Learning self-determination skills can help them concentrate on coursework and pursue the goals they desire [34,35]. Participants in the PATHS program were taught to monitor their course progress using a weekly report sheet to document their grades. Participants also needed to learn to manage their time to balance both coursework and extracurricular activities. In addition, through living independently, participants were able to make decisions and take responsibility for those decisions. They also needed to practice self-advocacy by knowing their rights and vocalizing their needs. These practices enhanced participants’ self-monitoring and self-management skills step by step.

However, regarding the third cohort, perhaps due to instructions being delivered through online learning, there were some drops in scores. Previous studies have documented the impact of the pandemic on education for students with disabilities. For example, Sheppard-Jones et al. [36] demonstrated that the pandemic may exacerbate disability-based disparities in learning outcomes; therefore, PSE programs should promote inclusive learning environments for those with disabilities. The impact of the sudden shift to online learning on education is already evident based on the score trend. The drops in scores implied that PSE programs should come up with an inclusive teaching model to address the disproportionate impact induced by the pandemic. Tecce DeCarlo et al. [24] revealed how to use technology to create an inclusive online learning environment. They showed that technology that mirrors the face-to-face experience and facilitates social interaction an help students and educators to cope with the challenges associated with the transition to online learning. Knowledge learned from the pandemic can be utilized in the future to establish an inclusive learning model for students with disabilities in PSE.

Regarding the employment outcomes, although we used social media and email to invite graduates to complete the follow-up survey, the pandemic made the recruitment process challenging. Only 12 graduates responded to the survey. Participation in the program seems to have a positive impact on students’ employment outcomes. Although this study had a limited sample size in terms of participants, with national employment rates for individuals with disabilities at less than 25% [37] and state-level employment rates lower than 10% [37], it is worth noting that 8 of the 12 graduates, who responded to the survey were employed and that all graduates had at least a semester of work experience during their time in the program. The PATHS program offers career-focused experiences. Participants in the program were placed in a practical setting to practice human service skills. In addition, of these eight graduates who were employed, five graduates were employed in areas related to their PATHS certification. They were hired as DSP or CCP to provide human services. It is evident that participation in the program was beneficial for participants to build up their desired career goals.

The global pandemic impacted work. Participants reported that the pandemic changed their work role or work hours due to school closures. Sinclair et al. [27] also reported that the pandemic impacted the work lives of people with IDD, reducing work hours and inducing jobs losses. Similarly, Jashinsky et al. [38] discussed the impact of the pandemic on the work lives of people with disabilities. They revealed that although technology allowed workers to do their jobs remotely, people with disabilities may still have challenges in completing their job tasks due to limited access to technology and a lack of training in using technology. Therefore, relevant stakeholders should continue to support people with IDD in securing a job during a national or global crisis. An employment-focused PSE program should not only provide employment skill training but also improve students’ abilities in using technology.

Lastly, when comparing all three cohorts, ethnicity, gender, and disability types were not found to have statistically different scores. However, these results could be impacted by the lack of homogeneity in the groups. Upon analyzing the scores for each cohort, we found that there were significant differences in scores based on gender, with females scoring higher than males in both the 2018–2019 and 2019–2020 cohorts. Similarly, individuals with high-incidence disabilities (e.g., SLD, ED) traditionally scored higher than peers with low-incidence disabilities (e.g., ID, Multiple Disabilities). The scores based on ethnicity varied significantly from cohort to cohort.

### 4.1. Implications for Research and Practice

The PATHS program is based on inclusion and training to prepare graduates for employment in specific career fields provide them with the skills required to work in multiple areas. The PATHS program does this through interventions that focus on skill acquisition, self-determination instruction, and independent living training. This research study aimed to focus on the impact of the interventions developed with this approach. However, additional research around the impact of the pandemic was conducted as well. From the results, it is clear that there is a positive correlation between participation in the PATHS program and skill acquisition and self-determination levels. The success of this approach is based on the many experiences that students have while enrolled in the program.

PATHS Program students are enrolled in school, take academic courses, receive bridge support for attending college, and live independently in an off-campus dorm-based environment. The PATHS Program also provides internships for students, within their communities while also delivering employment training before and during these internships. This means that students graduate with experience in their career fields and general employment skills experiences, as well as independent living training and daily skills training. Building programs that provide these opportunities are essential to increasing employment and post-secondary living opportunities for individuals with disabilities.

However, it is clear that additional research on graduate outcomes for these graduates, as well as the graduates of other programs, is necessary to understand the long-term impacts of post-secondary programs. Longitudinal research would allow for a better understanding of the successes of graduates, areas of need, and, in light of the pandemic, how external forces can interact to create significant challenges for future employment and living for graduates. Understanding how to better prepare students for future challenges would better support the adaptability of the graduates.

### 4.2. Limitations

There are several limitations that should be considered when exploring the relevance of this study for future application. First, the results of the third cohort were impacted by the pandemic; this means that the scores for the third cohort (2019–2020) were impacted during the spring term and impacted an initially positive trend. Second, as there was no homogeneity in groups regarding ethnicity, disability label, or gender, any statistical analysis needs to factor these in and would be more accurate with a larger sample size in each cohort. Third, although we attempted to collect employment data from all participants, graduates move and do not always keep in contact. While we did collect this data over months, it was not possible to reach out to all graduates. This means that for any statistically significant conclusions to be drawn around employment outcomes (both short- and long-term conclusions), a larger sample size is needed. Fourth, we acknowledge that it is impossible to remain completely objective when interpreting the findings. Researchers’ biases and preconceived notions might influence their discussions. While we tried to present the collected information as directly as possible, readers should be aware that researchers’ biases may have played a role in interpreting the findings.

## 5. Conclusions

Individuals with IDD enroll in PSE programs to receive training that aims to improve their self-determination, post-secondary readiness, and employment outcomes. However, little empirical research has been conducted to explore the impact of PSE programs on the acquisition of these skills for individuals with IDD. The findings from this study indicate that self-determination and post-secondary readiness among PATHS students showed a positive trend across all three cohorts. This research underscores the need for additional studies to determine whether PSE programs truly support skill acquisition for individuals with IDD. The findings enhance our understanding of the significant role PSE programs play in supporting individuals with IDD.

## Figures and Tables

**Table 1:** Participant characteristics.

Cohort	Cohort 1 (2017–2018) *N* = 16	Cohort 2 (2018–2019) *N* = 17	Cohort 3 (2019–2020) *N* = 15
Gender			
Female	9	14	10
Male	7	3	5
Race			
White	7	11	10
Hispanic	3	3	3
African American	3	0	0
Asian	1	1	1
More than one race	2	1	1
Unknown	1	1	0
Disability type			
ID	4	5	8
OHI	4	4	2
VI	2	2	1
TBI	2	0	0
SLD	2	1	2
EBD	2	0	0
HI	0	2	1
ASD	0	2	1
No identified disability	1	1	0

## Data Availability

Data available will only be made available upon request due to privacy restrictions.
